# The Management of Tibial Spine Avulsion Fracture in a Skeletally Immature Patient by Using Staple Fixation: A Case Report

**DOI:** 10.7759/cureus.102958

**Published:** 2026-02-04

**Authors:** Hafid Talha, Abdelkhalek Hamoutahra

**Affiliations:** 1 Pediatric Surgery, Moulay Ali Cherif Regional Hospital Center, Laboratory of Health Sciences, Faculty of Medicine and Pharmacy of Errachidia, Moulay Ismail University of Meknes, Errachidia, MAR; 2 Pediatric Surgery, Faculty of Medicine and Pharmacy, Moulay Hassan Ben Mahdi Regional Hospital, Laayoune, MAR

**Keywords:** anterior cruciate ligament avulsion, pediatric knee trauma, skeletally immature patient, surgical fixation, tibial spine avulsion fracture

## Abstract

Tibial spine (TS) fractures are uncommon injuries in children and typically occur after sports-related trauma or road traffic accidents. We report the case of an 11-year-old male who presented with severe pain, swelling, and an inability to move the left knee after a direct impact during a road traffic accident. Initial radiographs demonstrated a displaced TS fracture. CT confirmed an avulsion fracture at the anterior cruciate ligament (ACL) tibial insertion with intra-articular incarceration of the fragment. MRI showed bone contusions and an avulsed ACL insertion without disruption of the ACL fibers. Given the complexity of the displaced fracture pattern, surgical treatment was performed. Open reduction and internal fixation were performed using permanent staples positioned to avoid crossing the physis, and intraoperative imaging confirmed anatomic reduction and stable fixation. At the three-month follow-up, radiographs demonstrated satisfactory healing with a clinically stable knee. This report highlights a physeal-sparing fixation strategy employing staples for a complex pediatric TS fracture.

## Introduction

Fractures of the tibial spine (TS) are uncommon in children, accounting for less than 5% of osseous knee injuries in the pediatric population [1]. They are most often reported after bicycle accidents or sports-related trauma in young athletes [2]. These injuries are more frequently observed in skeletally immature patients, in whom the anterior cruciate ligament (ACL) is relatively more resistant than the developing insertional structures of the proximal tibia [3]. Surgical management is generally considered the treatment of choice, particularly for displaced fractures or those associated with ACL avulsion [2]. Fixation must be meticulous to restore the extensor mechanism and reduce the risks of knee instability and nonunion. In skeletally immature patients, operative treatment can be challenging because standard hardware may violate or irritate the physis, potentially affecting limb growth. We report a case in which we performed open fixation using grafts to avoid disruption of the growth plate and minimize the risk of growth-related complications. At the three-month follow-up, the patient had a stable knee and satisfactory clinical and radiographic outcomes.

## Case presentation

**Figure 1:**
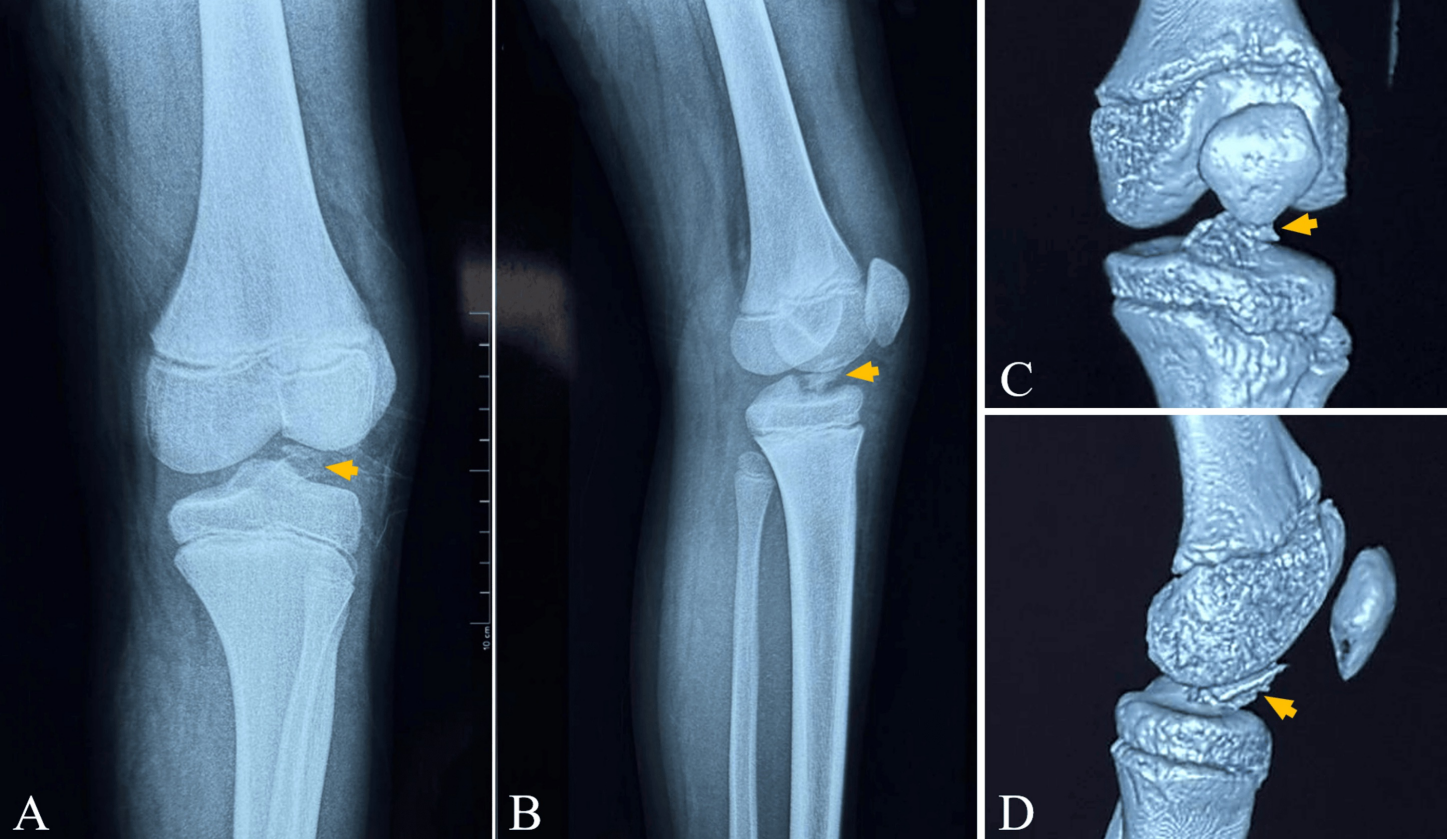
Plain radiograph and CT findings Initial plain radiographs show a displaced tibial spine fracture on the anteroposterior (A) and lateral (B) views. Three-dimensional CT reconstructions demonstrate the avulsed fragment in en face (C) and lateral (D) views CT: computed tomography

The patient was an 11-year-old male with no significant past medical history who presented to the emergency department after a road traffic accident with a direct impact to the left knee. Immediately after the injury, he developed severe pain and complete functional limitation of the left knee, with an inability to move the joint. On admission, he was clinically stable with normal vital signs (heart rate: 85 beats/min, respiratory rate: 18 breaths/min, blood pressure: 105/60 mmHg, and oxygen saturation: 98% on room air). Examination of the left knee showed marked swelling and significant tenderness, with flexion and extension not possible due to pain and mechanical limitation. The remainder of the physical examination was unremarkable. Standard anteroposterior and lateral radiographs of the left knee demonstrated a displaced TS fracture (Figure 1). CT of the knee with multiplanar and 3D reconstructions further characterized the injury, showing an avulsion fracture of the anterior TS at the ACL insertion. The osseous fragment was displaced and elevated, with intra-articular incarceration between the femorotibial articular surfaces (Figure 1).

**Figure 2:**
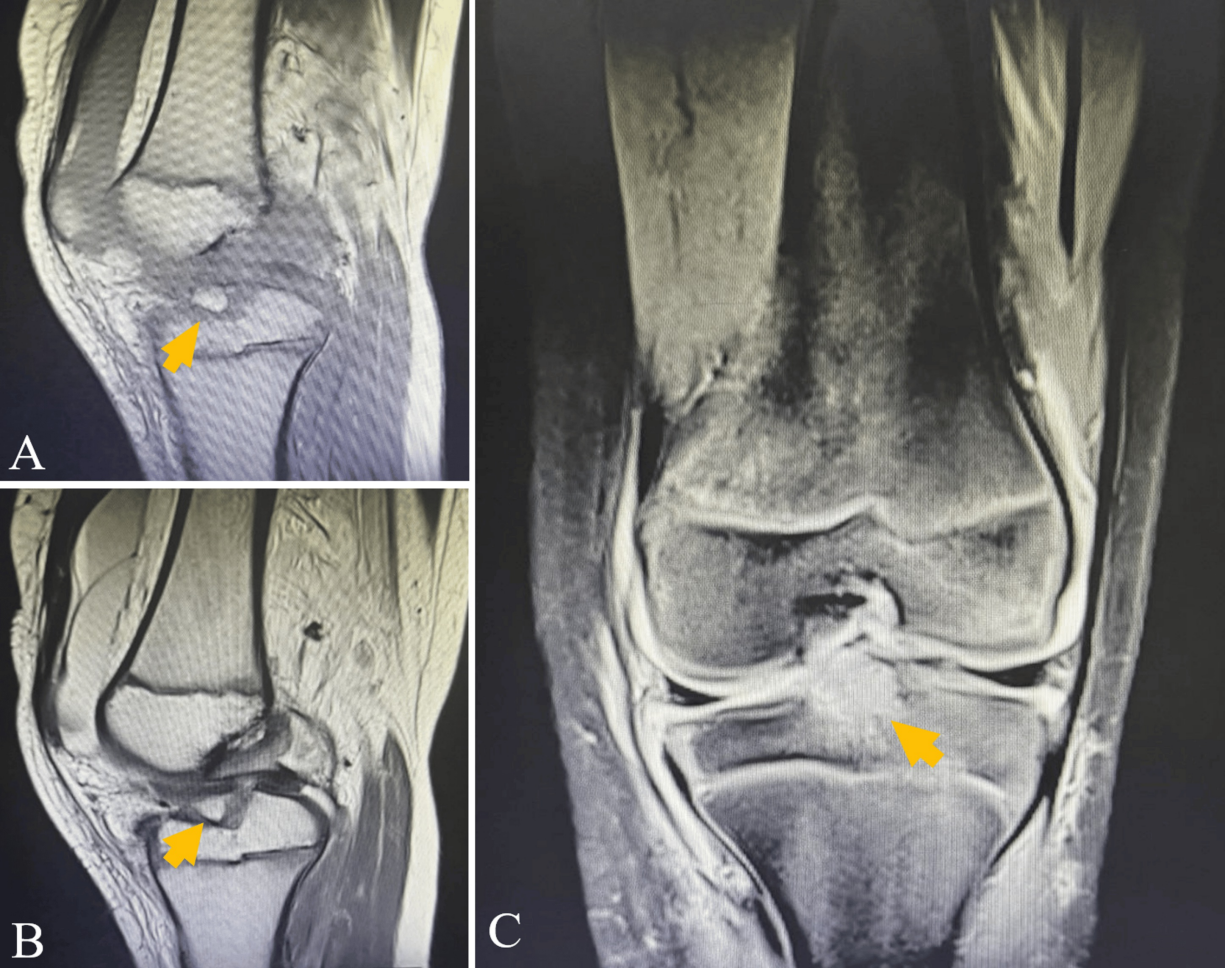
MRI findings MRI (T2 fat-suppressed/STIR) demonstrates bone contusions (arrow) as a hyperintense marrow signal and an avulsion of the ACL tibial insertion with elevation of the anterior tibial eminence, without disruption of the ACL fibers, on sagittal (A, B) and coronal (C) images MRI: magnetic resonance imaging; STIR: short TI inversion recovery; ACL: anterior cruciate ligament

MRI provided additional soft-tissue assessment. Bone contusions were noted as hyperintense areas on T2 fat-suppressed/STIR sequences. The ACL insertion was avulsed, with elevation of the anterior margin of the tibial eminence, without evidence of ACL fiber disruption or meniscal lesions (Figure 2).

**Figure 3:**
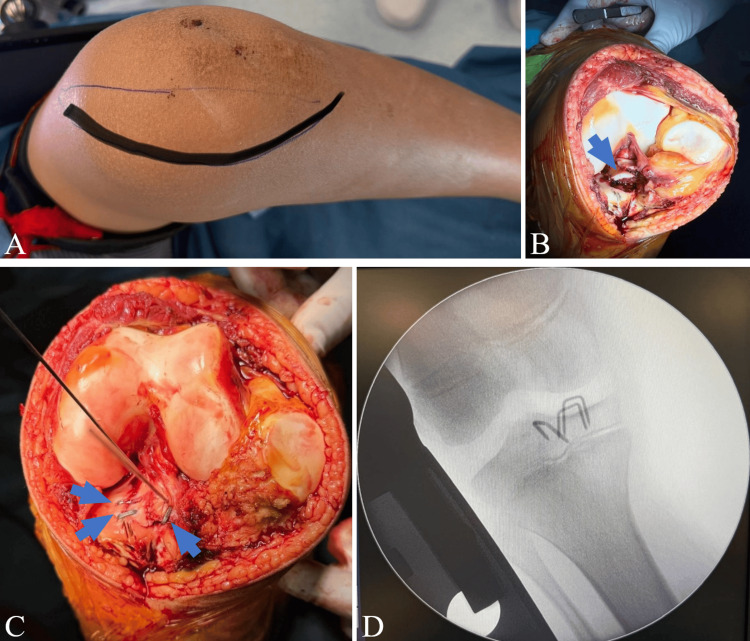
Intraoperative photographs The images show the medial surgical access (A), the tibial eminence fracture fragment (arrow) before fixation (B), and after fixation with three staples (arrows) (C), with fluoroscopic confirmation of staple position (D)

**Figure 4:**
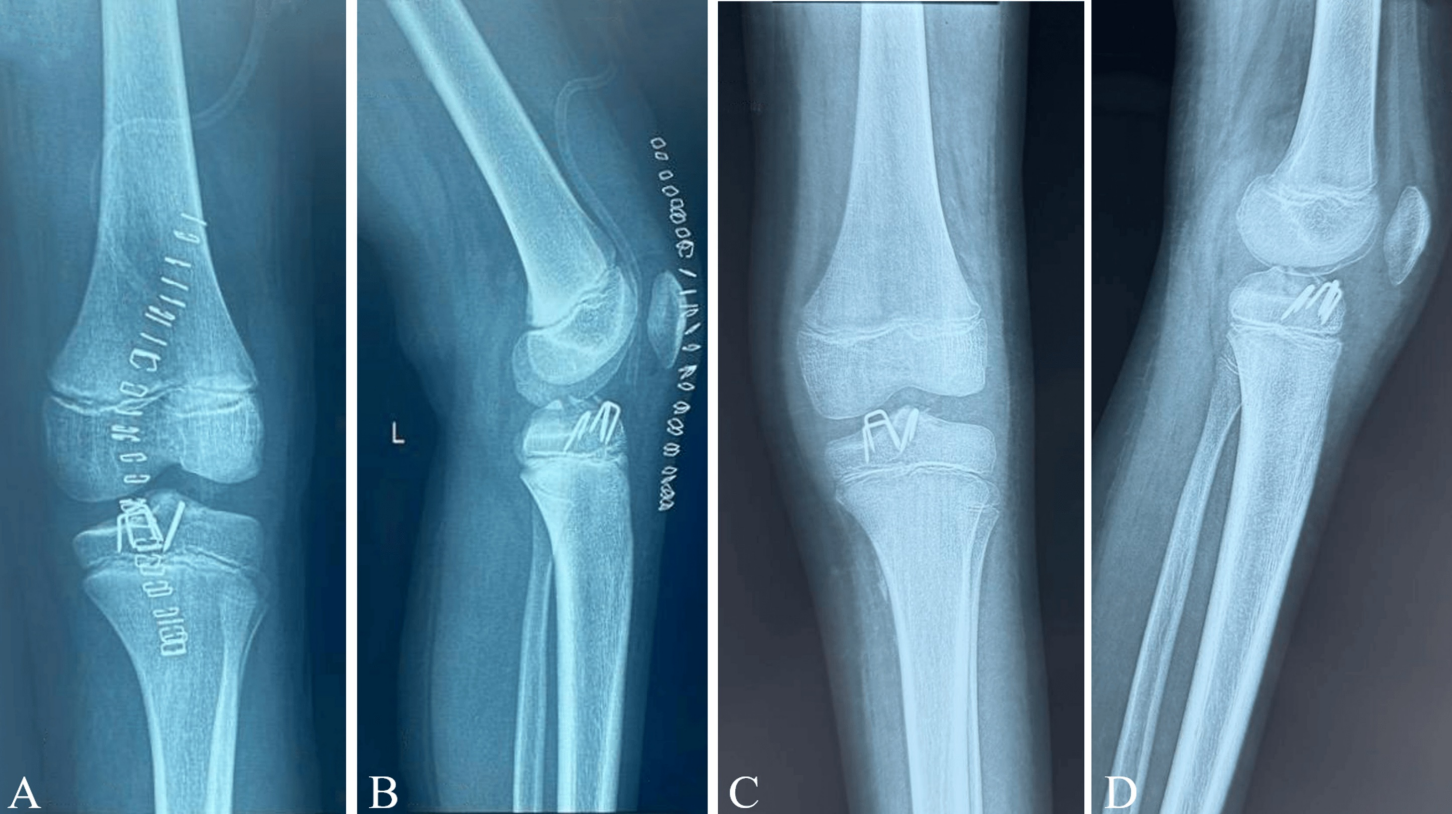
Immediate postoperative and three-month follow-up radiographs Immediate postoperative radiographs demonstrate staple fixation of the tibial spine on the anteroposterior (A) and lateral (B) views. Three-month follow-up radiographs show stable fixation on the anteroposterior (C) and lateral (D) views

Surgical treatment was performed. Just before the procedure, a Lachman test was performed, which was positive, demonstrating increased anterior tibial translation compared with the contralateral knee, consistent with ACL avulsion. Intraoperative findings confirmed a complex tibial eminence fracture consisting of two fragments. Reduction and fixation were achieved using staples placed without crossing the physis. Intraoperative fluoroscopy confirmed anatomic reduction and stable fixation, with the staples remaining proximal to the physeal cartilage (Figure 3). Immediate postoperative radiographs demonstrate staple fixation of the TS. At the three-month follow-up, radiographs demonstrated satisfactory healing of the tibial eminence fracture (Figure 4).

## Discussion

TS fractures are uncommon in children and adolescents, accounting for approximately 2 to 5% of all knee fractures [4]. They occur most often between the ages of eight and 14 years [5]. These injuries are classically associated with bicycle accidents and youth sports, such as soccer and rugby, with most cases sharing a common mechanism involving knee hyperflexion with the tibia in external rotation [6]. The same mechanism may cause ACL rupture; however, in skeletally immature patients, the relative weakness of the ACL tibial insertion compared with the ligament itself predisposes patients to avulsion of the TS rather than to midsubstance ACL rupture [2]. TS fractures may also result from direct trauma or knee hyperextension [7].

Severity is commonly classified using the modified Meyers and McKeever system, which describes nondisplaced fractures (Type I), anteriorly displaced fractures (Type II), completely displaced fractures (Type III), and Type IV fractures, which were later described by Zaricznyj and correspond to completely displaced comminuted fractures [1]. Clinically, patients typically present with knee pain and swelling [2]. Plain radiographs may demonstrate the fracture; CT can be particularly helpful in defining the fracture pattern and displacement in complex cases, while MRI is useful for evaluating associated soft-tissue injuries, especially meniscal and ligamentous lesions [2].

Nonoperative management is guided by the Meyers and McKeever classification. Isolated Type I fractures are typically treated with cast or splint immobilization and generally have favorable long-term outcomes [8]. Selected Type II fractures may also be treated nonoperatively after closed reduction with radiographic confirmation [2]. Immobilization is usually achieved with a long-leg cast or brace, often in approximately 20° of knee flexion to reduce tension on the ACL [9].

Operative management is generally recommended for displaced TS fractures, and Type III and Type IV injuries are almost universally treated surgically [10]. Compared with nonoperative care, surgery has been associated with better functional outcomes and higher patient satisfaction, with lower reported complication rates in the literature [2]. Open reduction and internal fixation and arthroscopic reduction and internal fixation have shown broadly similar outcomes, with no major differences reported in knee stability or postoperative measures in some comparative data. In contrast, a pediatric systematic review reported greater range-of-motion deficits and increased laxity on Lachman testing after arthroscopic procedures compared with open procedures [11]. In practice, both approaches are acceptable and should be chosen based on the technique that can be performed most efficiently for the patient and operating team.

A wide variety of implants may be used for fixation, including screws (traditional, headless, and cannulated), wires such as Kirschner wires, and staples [2]. Screw fixation is often favored for its simplicity and perceived reliability, and some studies, particularly those including adults, have reported better short-term outcomes with screws. Cannulated screws may allow shorter immobilization and earlier weight-bearing than suture fixation in some reviews [12]. However, in skeletally immature patients, metal screws may be associated with physeal injury (including growth arrest) and articular surface damage. Headless screws and Kirschner wires have been reported to reduce intra-articular prominence and the need for later hardware removal [13]. Suture-based techniques may provide stronger and more durable fixation under cyclic loading in biomechanical studies. Other less common methods, such as suture pullout fixation, cortical button constructs, hybrid techniques, and tension-band methods, have also been reported with success in selected series [2].

In our case, the decision to proceed with an open approach was primarily driven by the complex Type IV fracture configuration according to the Meyers and McKeever classification and the need for direct operative assessment of the injury. We chose permanent staples because they could be positioned so as not to cross the growth plate, thereby minimizing the risk of physeal injury and subsequent limb growth disturbance. In a series by Sundararajan et al., 22 patients aged 15 to 55 years underwent arthroscopic fixation using one or two staples and showed good postoperative scores, with only one patient experiencing a backed-out staple, which was removed [14]. Staple fixation is also technically straightforward and may avoid drilling, tapping, or the insertion of long screws that can be required with other tools.

Complications of TS fractures include arthrofibrosis, the most common complication, resulting in postoperative knee stiffness. Residual ACL laxity, which is more frequent after nonoperative management, and, less commonly, nonunion, which is rare in children but may lead to instability, pain, and loss of motion, are often related to inadequate reduction or loss of fixation and are reported more frequently after nonoperative treatment of displaced fractures. Other reported complications include retropatellar knee pain, quadriceps atrophy, and tibial physeal disturbances [2].

Postoperative radiographs are used to confirm adequate reduction of the tibial spine fragment. The knee is typically immobilized in extension or slight flexion for four to six weeks. Weight-bearing protocols vary, ranging from delayed weight-bearing until six weeks postoperatively to earlier weight-bearing as tolerated [1].

## Conclusions

This report highlights that displaced TS fractures in children require prompt recognition and careful imaging evaluation to confirm the diagnosis and define associated soft-tissue injuries. In a skeletally immature patient with a complex fracture pattern, stable anatomic fixation that respects the physis is essential to restore knee stability and minimize the risk of growth-related complications. Physeal-sparing staple fixation provided reliable stabilization and satisfactory healing in our patient, making it a practical option in selected pediatric cases when meticulous reduction and appropriate postoperative rehabilitation are ensured. Larger-scale studies are needed to clarify the true clinical value of staple fixation compared with other fixation techniques and to determine whether it offers consistent advantages in clinical outcomes and complication rates in the pediatric population.
